# Serrated neoplasia in the colorectum: gut microbiota and molecular pathways

**DOI:** 10.1080/19490976.2020.1863135

**Published:** 2020-12-31

**Authors:** Xing Kang, Ru Zhang, Thomas Ny Kwong, Rashid Ns Lui, William Kk Wu, Joseph Jy Sung, Jun Yu, Sunny H Wong

**Affiliations:** a Department of Medicine and Therapeutics, Faculty of Medicine, The Chinese University of Hong Kong, Hong Kong SAR, China; b State Key Laboratory of Digestive Disease, Institute of Digestive Disease, Li Ka Shing Institute of Health Sciences, Faculty of Medicine, The Chinese University of Hong Kong, Hong Kong SAR, China; c Division of Gastroenterology, Department of Medicine, Shenzhen People’s Hospital, Shenzhen, China; d Department of Anaesthesia and Intensive Care, Faculty of Medicine, The Chinese University of Hong Kong, Hong Kong SAR, China; e Shenzhen Research Institute, The Chinese University of Hong Kong, Shenzhen, China

**Keywords:** Serrated pathway, microbiota, colorectal cancer, neoplasia

## Abstract

Colorectal cancer (CRC) is a heterogeneous disease with different gene expression patterns. There are two major colorectal carcinogenesis pathways: conventional adenoma-carcinoma pathway and alternative serrated neoplasia pathway. Apart from the conventional pathway that is typically initiated by characteristic *APC* mutation and chromosomal instability, the serrated neoplasia pathway is mainly characterized by mutations of *BRAF* or *KRAS*, microsatellite instability (MSI), and CpG island methylator phenotype (CIMP). Despite the malignant potential of serrated lesions, they can be easily overlooked during endoscopy screening and even in pathological assessment due to its anatomical location, morphology, and histological features. It has been shown that environmental factors especially the gut microbial composition play a key role in CRC pathogenesis. Thus, the preferential localization of serrated lesions in specific intestine areas suggest that niche-specific microbiota composition might intertwined with host genetic perturbations during the development of serrated lesions. Although serrated lesions and conventional adenomas are biologically different, most studies have focused on conventional adenomas, while the pathophysiology and role of microorganisms in the development of serrated lesions remain elusive. In this review, we discuss on the role of gut microbiota in the serrated neoplasia pathway of colorectal carcinogenesis and its specific clinical and molecular features, and summarize the potential mechanisms involved.

## Introduction

Colorectal cancer (CRC) is the third most common cancer and the second leading death of cancer worldwide.^1^ In 2018, CRC was the most commonly diagnosed gastrointestinal cancer, constituting 10.2% and 9.2% cancer cases and deaths respectively worldwide.^2^ In the United States, CRC is estimated to make up 8.2% and 8.8% of total cancer incidence and mortality in 2020, respectively.^3,4^ Malignant changes in the intestinal tract are often developed from a focal dysplastic polypoid precursor, the adenoma, which accumulates further genetic mutations and progresses following the adenoma-carcinoma sequence.^5^ Similar to conventional adenomas, serrated lesions in the colorectum have a potential to transform into malignant CRC,^6^ especially large serrated lesions that are located in the proximal colon.^7^

The development of CRC follows several distinct mechanistic pathways, including the adenoma–carcinoma pathway and serrated neoplasia pathway.^8^ While the conventional adenoma-carcinoma pathway is more common, a small subset of CRC occurs through the serrated pathway. In the past, these serrated lesions were considered as relatively benign lesions;^9^ however, emerging evidences suggested that certain sessile lesions are non-adenomatous precursors of malignant cancers.^10,11^ In the fifth edition of the World Health Organization classification of digestive tumors, sessile serrated polyp/adenoma was renamed as sessile-serrated lesion (SSL). In the British pathological classification system, serrated lesions can be classified into several lesion types, including hyperplastic polyp (HP), SSL, SSL with dysplasia, traditional-serrated adenoma (TSA) and mixed polyp.^10^ SSLs and TSAs have been recognized as important precancerous lesions of CRC.

Because of their indistinctive morphological and histological features, serrated lesions can be easily overlooked during colonoscopy and even in pathological assessment. SSLs are typically flat or sessile under endoscopic visualization, and are occasionally covered by a mucus cap.^10^ Many CRCs derived from SSLs are located in the right side of the colon, with molecular features of *BRAF* mutation, high microsatellite instability (MSI), and CpG island methylator phenotype (CIMP). These cancers are thought to account for a large proportion of interval cancers and may represent the main cause of cancer screening failure. Thus, it is important to study the serrated pathway to develop better management strategies for these cancers.

Various genetic and environmental factors contribute to colorectal carcinogenesis. Previous twin studies showed that the heritability of CRC is only around 12–35%,^12^ suggesting that environmental factors may play a greater role in sporadic CRC.^8^ Certain environmental factors are associated with serrated colorectal neoplasia. Systematic reviews found that smoking, alcohol, and body mass index were more strongly associated with serrated polyps than conventional adenomas.^13,14^ A strong association between red meat consumption and risk of SSLs was also shown in a colonoscopy-based case–control study.^15^ These epidemiological findings could enhance our mechanistic understanding and help identify mitigating strategies for serrated neoplasia.

Furthermore, the microbiota has recently received increasing attention as a non-genetic factor in colorectal neoplasia. Tens of trillion microorganisms colonize the human gastrointestinal tract,^16^ to interact with our epithelial cell as part of the host–microbe interaction.^17,18^ Research in recent years showed that several bacteria is associated with CRC, including *Fusobacterium nucleatum, Bacteroides fragilis*, and other CRC-enriched bacteria,^19^ through different pro-inflammatory and pro-carcinogenic mechanisms.^20^ Despite this, the role of gut microbiota in the serrated neoplasia remains largely unknown.

In this article, we review the role of microbiota and molecular pathways pertinent to the formation of serrated neoplasm.

## The serrated neoplasia pathway

Our knowledge on the molecular pathways of colorectal adenomas and other precancerous lesions has increased substantially over the past few years. With the advent of molecular testing for MSI, *RAS* (*KRAS, NRAS)* and *BRAF* mutations, accurate and tailored treatment for advanced CRC is possible.^21^ These tumor genetic insights have shed light on their precursor lesions as well. There are two main pathways of carcinogenesis: the conventional adenoma-carcinoma pathway (also known as chromosomal instability pathway) and the alternative serrated neoplasia pathway.^22^ Conventional adenomas are typically initiated by *APC* mutations, followed by *RAS* activation or loss of function mutations in *TP53*.^22^ In contrast, the serrated neoplasia pathway is mainly characterized by mutations of *BRAF* or *KRAS*, chromosomal stability, and CIMP.^22^ Most CRC develop through the conventional adenoma-carcinoma pathway, while approximately 10–20% of CRC cases occur through the alternative serrated neoplasia pathway.^22^ Autopsy studies showed that the prevalence of serrated lesion varies, but in general about 25% of adults have one or more serrated lesions.^23^ Recently, a systematic review identified 74 relevant colonoscopy studies and found that SSL prevalence greatly varied by geographical regions, ranging from 2.6% in Asia to 10.5% in Australia.^24^

**Figure 1.**
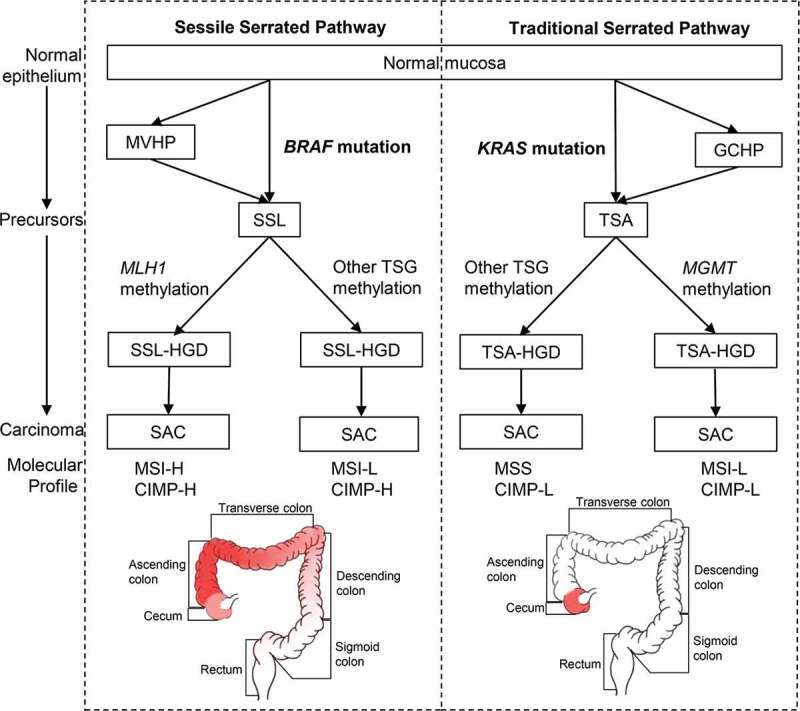
The sessile (left) and traditional (right) serrated pathways. Frequently affected areas for colorectal tumors in each pathway are highlighted in red and the color depth represents the frequency of CIMP-H, MSI-H and *BRAF*/*KRAS* mutations in CRC. Abbreviations: MVHP, microvascular hyperplastic polyp; GCHP, goblet cell-rich hyperplastic polyps; SSL, sessile serrated lesion; TSA, traditional-serrated adenoma; *MLH1, MutL homolog 1; MGMT*, O-6-methylguanine-DNA methyltransferase; TSG, tumor suppressor genes; SSL-HGD, sessile serrated lesion with high-grade dysplasia; TSA-HGD, traditional-serrated adenoma with high-grade dysplasia; SAC, serrated adenocarcinoma; MSI-H, high-level microsatellite instability; MSI-L, low-level microsatellite instability; MSS, microsatellite stability; CIMP-H, high-level CpG island methylator phenotype; CIMP-L, low-level CpG island methylator phenotype

In 2007, Makinen evaluated three molecular alterations to help further subtype serrated lesions.^25^ By combining the *RAS* mutations, the degree of MSI, and the level of CIMP, two separate serrated pathways^26^ could be classified:^11,27^ (1) Sessile serrated pathway with *BRAF* mutation, MSI-H/L and CIMP-H, typical lesions being SSLs, and (2) Traditional serrated pathway with *KRAS* mutation, low-level MSI (MSI-L) or microsatellite stability (MSS), and CIMP-L, typical lesions being TSAs (Figure 1).

Further studies have investigated the anatomical locations of these colorectal lesions. Although Bufill et al. divided the colorectal tumor location at splenic flexure into proximal and distal colons in 1990,^28^ the frequencies of the molecular signatures, including CIMP-H, high-level MSI (MSI-H), and *BRAF* mutations do not change abruptly at the splenic flexure.^29^ Instead, these frequencies increased gradually from the rectum to ascending colon, followed by a relatively decrease in the cecum,^29^ challenging the common conception of discrete molecular features of proximal (right-sided) versus distal (left-sided) CRC^30,31^ (Figure 1). Nevertheless, cecal cancers harbor a high frequency of *KRAS* mutations.^29^

## Consensus molecular subtypes (CMSs)

**Figure 2.**
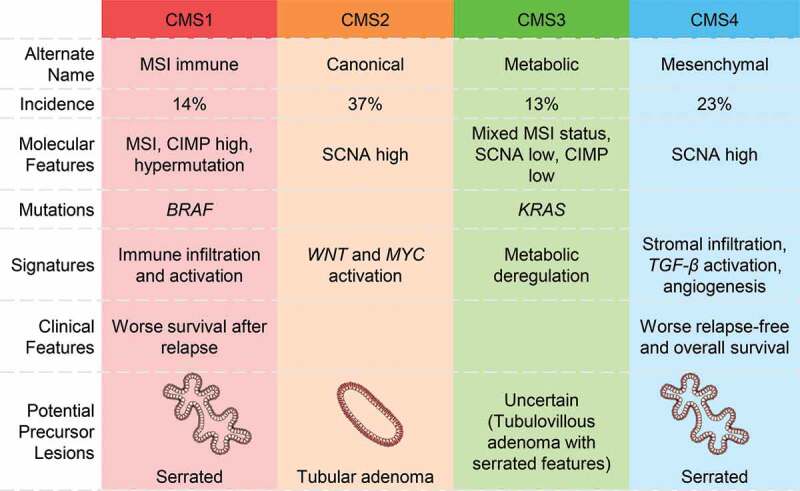
Consensus molecular subtypes (CMS) in CRC and their precursor lesions. Abbreviations: MSI, microsatellite instability; CIMP, CpG island methylator phenotype; SCNA, somatic copy number alterations

CRC is a heterogeneous disease with distinctive gene expression patterns.^32–38^ In the genomic analysis of 276 samples in the Cancer Genome Atlas Project, three-quarters among the hypermutated tumors had high MSI, usually with hypermethylation and *MLH1* silencing, were located in the right colon and were frequently associated with CIMP.^38^ Schlicker et al. first reported an epithelial-mesenchymal-transition (EMT) expression signature defined subgroup in 2012.^34^ Subsequent molecular classifications of CRCs based on its stemness, *Wnt* pathway expression,^35^ and clinicopathological features^36^ have been proposed. Marisa et al. identified six molecular subtypes associated with distinct clinicopathological characteristics, molecular alterations, specific enrichments of supervised gene expression signatures (stem cell phenotype-like, normal-like, serrated colon cancer phenotype-like), and deregulated signaling pathways.^37^ Budinska et al. distinguished five different gene expression CRC subtypes, which are surface crypt-like, lower crypt-like, CIMP-H-like, mesenchymal, and mixed.^32^ A molecular classification associated with prognosis and chemotherapy response was developed by Roepman et al. in 2014, which consist of three major intrinsic subtypes (A-, B- and C-type) based on three tumor biological hallmarks: *EMT*, mismatch repair genes deficiency, and cellular proliferation.^33^ To better consolidate the biological findings and enhance international communications, the consensus molecular subtypes (CMS) was proposed in 2015 to unify six independent transcriptome-based CRC subtyping strategies as abovementioned.^32–37,39^ The four subtypes with distinguishing features include: CMS1 (MSI immune) tumors that are immunogenic, microsatellite unstable, and hyper-mutated; CMS2 (canonical) tumors that show *WNT* and *MYC* signaling activation; CMS3 (metabolic) tumors that have metabolic dysregulation; and CMS4 (mesenchymal) tumors that have stromal infiltration, TGF-β activation, angiogenesis^39^ (Figure 2). Samples with mixed features are transition phenotypes or may represent intra-tumoral heterogeneity.

This molecular scheme raised an immediate question to how the pathological precursor types are related to the cancer subtypes. To address this question, Fessler et al. investigated the role of premalignant lesions using organoid culture and found that SSLs overexpressed *TGF-β* signaling, a key molecular characteristic of CMS4 subtype of CRC.^40^ Besides, Chang et al. analyzed the transcriptomes of 311 sporadic and 78 hereditary adenomatous and serrated lesions by a random forest classifier, and found that adenomatous polyps showed a highly similar transcriptomic profile to the CMS2 subtype, whereas the transcriptomic profiles of HP and SSL resemble that of the CMS1 subtype. Together with their right-sided anatomic location and *BRAF* mutations,^41^ this suggests a strong relationship between serrated lesions and the CMS1 subtype of CRC. Nevertheless, significant *KRAS* mutations were not observed probably because of the small number of precursor lesions resembling CMS3 in their study. The relationships between premalignant lesions (SSLs versus tubular adenomas^42^) and CMS3 tumors^42,43^ remain uncertain. Furthermore, a recent systematic review suggested tubulovillous adenomas with serrated features to be precursors of *KRAS* mutant tumors.^44^ Tsai et al. evaluated the pathological and molecular features of 60 TSAs with cytologic dysplasia and/or invasive carcinoma, and shown that tubulovillous adenoma with serrated features had higher frequencies of *KRAS* mutations than TSAs with serrated dysplasia.^44,45^ Potential precursor lesions assigned to the CMSs based on the above research results are shown in Figure 2.

## Gut microbiota in serrated lesions

Recent literature has provided evidence that microorganisms can promote colorectal carcinogenesis.^20^ Nevertheless, these studies have focused on CRC and premalignant polyps derived from the conventional pathway,^20^ and the role of microorganisms in the serrated neoplasia is less clear. Peters et al. compared the stool microbiota between conventional adenoma and serrated lesions of 540 colonoscopy-screened adults by 16S rDNA gene sequencing and observed a significant depletion of *Erysipelotrichi* in 33 SSL cases.^46^ The increase of this bacterial class is associated with impenetrable mucus layer in mice^47^ and may play a protective role in SSL development. However, in a study from Iran, researchers analyzed the changes of fecal microbiota in patients with different precursor lesions including serrated lesions (21 HP and 16 SSL cases) and failed to observe significant differences in the microbiota.^48^ Similarly, a Korean study did not identify significant microbiota changes in rectal mucosae from healthy controls and patients with conventional adenoma, SSL, and CRC, respectively.^49^ However, both studies were limited by their small sample size. Thus, further studies with more samples could provide insight into the metagenomic landscape of SSLs.

There is a close association between *F. nucleatum* and CRC progression,^50^ and high level of *F. nucleatum* was associated with poor survival in metastatic CRC.^51^ Yu et al. examined the invasive *F. nucleatum* using 16S rRNA fluorescence in situ hybridization (FISH) and observed significantly more invasive *F. nucleatum* in proximal HPs and SSLs than that of conventional adenomas.^52^ On the contrary, Ito et al. detected *F. nucleatum* by quantitative PCR in HPs, SSLs, TSAs, and non-serrated adenomas, and found that this bacterium was not significantly associated with lesion histology, but rather was associated with right-sided premalignant lesions with *BRAF* mutation, CIMP-high, and MSI.^53^ Because of these features pointing to serrated neoplasia,^11,27^ the existence of colorectal *F. nucleatum* may influence CRC progression through serrated pathway. Another similar study by Park et al. compared the gut microbiota between tubular adenoma (TA) and SSLs and found that the relative abundance of *Fusobacteria* did not differ significantly between these patients.^54^ These two similar results suggested that *Fusobacteria* may contribute to carcinogenesis regardless of the molecular pathway.^53,54^ However, the small sample sizes and lack of multi-omics platforms have again limited these studies.

Furthermore, a study has associated CRC microbiota with tumor CMS type and identified some bacterial species specific to CMS1^55^ characterized by MSI and immune activation.^39^ Given the connection between CMS1 and serrated neoplasia,^41^ these species might contribute to the serrated pathway of CRC development. In this study,^55^ 16S rRNA analysis showed that the relative abundances of *Fusobacteria* and *Bacteroidetes* increased and the levels of *Firmicutes* and *Proteobacteria* decreased in CMS1. Species-level analysis showed that *Fusobacterium hwasookii* and *Porphyromonas gingivalis* are the most highly enriched species associated with CMS1, as well as oral pathogens such as *F. nucleatum, Parvimonas micra*, and *Peptostreptococcus stomatis*.

Lastly, there was a case report that human intestinal spirochetosis may be responsible for colonic adenomas or HPs.^4^ In a retrospective case–control study, the rate of human intestinal spirochetosis infection was significantly higher in SSL at 52.6% (10/19) compared to controls at 8.1% (14/172), which suggested a possible association between human intestinal spirochetosis and SSL.^56^ Nevertheless, this finding is yet to be validated in larger studies preferably from more diverse populations.

## Gut microbiota and specific molecular features

Many studies explored the microbial community of CRC samples in different cohorts, and established the associations of *F. nucleatum* with important clinical and molecular features.^53,57–63^ For instance, *F. nucleatum* was shown to be significantly associated with *MLH1* methylation,^53,57,59,60,63^ high-level MSI,^53,57,59–63^ high-level CIMP^53,59,60,63^ and *BRAF* mutation^57,59,61,62^ (Table 1). However, controversial data have been reported on whether *KRAS* mutations associated with *F. nucleatum* abundance.^53,58–65^ In a Brazilian study analyzing 43 fresh CRC tissues by qPCR and direct sequencing, Proenca et al. found that *KRAS* mutations occurred more frequently in *F. nucleatum*-infected CRC.^64^ Yamaoka et al. measured *F. nucleatum* copy numbers by droplet digital PCR and found a significant correlation between *F. nucleatum* abundance and *KRAS* mutations.^65^ Higher abundance of intra-tumoral *F. nucleatum* was also reported in CRC with proximal tumor location,^57,59,60^ higher clinical stage (T3/T4),^57,59,60^ poorer tumor differentiation,^57,59,60^ and worse survival.^57,59,66^ In addition, CIMP high cases were characterized by a high rate of mutations in MSI, *BRAF*^67^ and chromatin regulator genes, especially *CHD7* and *CHD8*,^68^ and rarely *KRAS* and *TP53* mutations.^67^ *F. nucleatum* abundance was found to be associated with *CHF7/8* mutation and *TP53* wild-type status.^63^ *KRAS* mutation was also detected, but there was no statistical difference between the mutation state and *F. nucleatum* abundance.^53,58–63^

**Table 1.** Serrated pathways associated with molecular features in *Fusobacterium nucleatum (Fn)* high expression CRC tissues. + indicates *Fn*-high CRC tissues exhibiting more frequent molecular features than Fn-low/negative ones (*P *< .05); whereas – indicates no significant difference of serrated pathway associated molecular features between Fn-high and Fn-low/negative tissues. Abbreviations: FFPE, formalin-fixed paraffin-embedded; Fn-high, high amount of *Fusobacterium nucleatum* DNA in tissues; Fn-low, low amount of *Fusobacterium nucleatum* DNA in tissues; MSI-H, high-level microsatellite instability; CIMP-H, high-level CpG island methylator phenotype

Authors	Year	Cohort	Specimen Type	Detection Method	Molecular Features in Fn-high Tissues				
					MLH1 Methylated	MSI-H	CIMP-H		BRAF Mutation
Tahara et al.^[63]^	2014	United States	Fresh-frozen tissue	qPCR	+	+	+	-	
Ito et al.^[53]^	2015	Japanese	FFPE tissue	qPCR	+	+	+	-	
Mima et al.^[60]^	2015	United States	FFPE tissue	qPCR	+	+	+	-	
Mima et al.^[59]^	2016	United States	FFPE tissue	qPCR	+	+	+	+	
Nosho et al.^[61]^	2016	Japanese	FFPE tissue	qPCR	/	+	/	+	
Park et al.^[62]^	2017	Korean	FFPE tissue	qPCR	-	+	-	+	
de Carvalho et al.^[57]^	2019	Brazilian	Fresh-frozen tissue	16S rDNA sequencing, qPCR	+	+	/	+	

Besides *F. nucleatum*, correlations between other microbial species with the status of *MLH1, BRAF, KRAS* were also reported. Immunohistochemical analysis indicated that *KRAS* and *BRAF* expressions were obvious in tumor with high abundance of *F. nucleatum* and *Bacteroides fragilis*, while tumors with *MLH1* mutation showed lower abundance of these species.^66^ Moreover, a high abundance of *F. nucleatum* and *B. fragilis* were independent indicators of poor survival.^66^ A positive correlation between *Ruminococcus gnavus* and *KRAS* mutation in aberrant crypt foci samples was also described, although this finding was only reported in one study with a limited sample size.^69^ As described previously, serrated neoplasia is characterized by high *MLH1* deficiency, *KRAS* and *BRAF* mutation,^6,11,25,27^ yet the association with *F. nucleatum, B. fragilis*, or *R. gnavus* remains unclear and needs to be explored in future studies.

## Potential mechanisms of microbial dysbiosis in serrated neoplasm formation

The fact that serrated lesions are preferentially localized in specific colonic locations^43^ suggested that non-genetic factors, such as niche-specific microbiota, may interplay with genetic perturbations to affect their development. To verify this hypothesis, Lira et al. have modeled a series of transgenic mice.^70–73^ Based on the immunohistochemical and immunoblot analyses, they found that the *EGFR* signaling pathway is activated in human-serrated lesions.^70^ Activation of *EGFR* signaling by transgenic expression of the *EGFR* ligand *heparin-binding epidermal growth factor-like growth factor (HBEGF)* in mice intestine promotes the development of cecal-serrated lesions.^70^ It showed that host-specific microbiota was associated with serrated polys, and microbiota alteration induced by antibiotics or by embryo transfer rederivation suppressed the formation of serrated lesions in the cecum of *HBEGF* transgenic mice.^72^ The development of serrated lesions was associated with epithelial barrier breakdown, bacterial invasion, and overexpression of several inflammatory factors.^72,73^ The release of IL1B from inflammatory macrophages stimulate subsets of cecal *platelet-derived growth factor receptor alpha+ (PDGRFA+)* fibroblasts during an early stage of serrated lesion development, resulting in upregulation of *Matrix Metallopeptidase 3 (MMP3)*, which can promote inflammation and accelerate serrated lesion development by facilitating *HBEGF/EGFR* signaling.^73^ Using 16S rDNA sequencing, the authors showed that the bacterial phylum of *Verrucomicrobia* was enriched, whereas *Deferribacteres* was decreased in the mouse cecal mucosa of serrated lesions compared to rederived HBUS mice.^72^

As discussed previously, *F. nucleatum* is an important bacterium in CRC and shows association with serrated neoplasia. *F. nucleatum* attaches and invades human epithelial cells via adhesion (FadA).^74^ Another virulence factor from *F. nucleatum*, an autotransporter protein (Fap2), has been shown to promote CRC progression by suppressing immune cell activity.^75^ Kostic et al. reported that *F. nucleatum* selectively recruits myeloid-derived immune cells (MDSCs) in CRC.^76^ *F. nucleatum* increases the production of reactive oxygen species (ROS), ^76,77^ possibly by MDSCs recruit. Tumor-associated MDSCs promote carcinogenesis through oxidative metabolism, including the production of ROS in human CRC.^78^ ROS induction is correlated with DNA methylation.^79^ Interestingly, methylation could also occur in promoter regions of *MLH1* gene and lead to MSI,^61,80^ which are the characters of sessile-serrated pathway.

Another mechanism for serrated neoplaia progression related to *F. nucleatum* is a tumor immunosuppressive microenvironment. *F. nucleatum* is associated with a lower density of CD3 + T cells in a US cohort,^60^ and *F. nucleatum* high MSI-H CRC was significantly associated with a high density of CD68+ tumor-infiltrating macrophages, a special subtype of MDSC.^62^ A study by Hamada et al. found that the presence of *F. nucleatum* in CRC tissues was associated with MSI, lower-level tumor-infiltrating lymphocytes (TIL), and poor clinical outcomes.^81^ Therefore, *F. nucleatum* may promote immune evasion by suppressing anti-tumor immune responses in MSI-H CRC. Moreover, the *F. nucleatum* derived FadA can interact with *E-cadherin* to promote CRC cells proliferation.^74^ This may be relevant to serrated lesions, as altered expression and localization of E-cadherins and its associated β-catenin have been described in hyperplastic polyps and serrated adenomas.^82^ The change in E-cadherin expression may be related to epithelial remodeling and stratification implicated in serrated adenoma formation.

**Figure 3.**
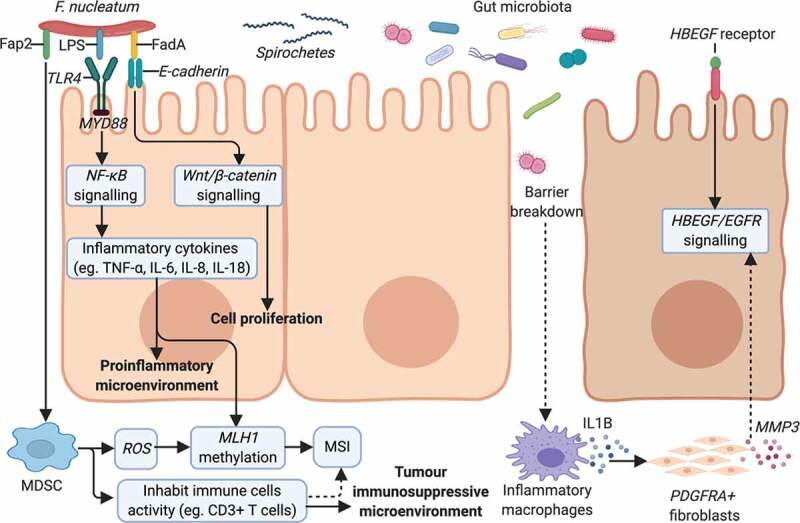
Potential mechanisms of gut microbiota dysbiosis on serrated neoplasm formation. *F. nucleatum* presents the virulence factors of FadA,^74^ Fap2^75^ and LPS,^64^ mediating its invasion and the promotion of serrated tumors. *F. nucleatum* can increase cell proliferation by binding of FadA^74^ to *E-cadherin* to activate the *Wnt/β-catenin* pathway.^74^ The *TLR4/MYD88* pathway is stimulated in response to LPS on *F. nucleatum*,^64^ activating *NF-κB*^64^ and resulting in a pro-inflammatory microenvironment.^64,66,74,76,83,84^ *F. nucleatum* modifies the tumor microenvironment by attracting MDSC^76^ and suppressing anti-tumoral immune responses.^60,81^ MDSCs can produce *ROS*,^76–78^ inducing *MLH1* methylation^79^ and leading to MSI.^61,80^ Other microorganisms, like spirochetes,^4,56^ may also participate in the serrated pathway of cancer formation. *EGFR* signaling activation was observed in human-serrated polyps^70^ and the role of gut microbiota was confirmed in transgenic HBUS mice.^72,73^ Subsets of cecal *PDGFRA+* fibroblasts are activated by IL1B released from inflammatory macrophages during an early stage of serrated lesions development.^73^ Proinflammatory genes and *MMP3* are upregulated in activated fibroblasts, which can promote inflammation and SP development by facilitating *HBEGF/EGFR* signaling.^73^ Abbreviations: Fap2, *F. nucleatum* autotransporter protein 2; LPS, lipopolysaccharide; FadA, *F. nucleatum* adhesin; *NF-κB, nuclear factor-κB*; MDSC, myeloid-derived immune cell; *ROS, reactive oxygen species; MLH1, mutL homolog 1*; MSI, microsatellite instability; *EGFR, epidermal growth factor receptor; HBEGF, heparin-binding epidermal growth factor-like growth factor; PDGRFA+, platelet-derived growth factor receptor alphaþ positive; MMP3, matrix metallopeptidase 3.*

Finally, *F. nucleatum* can also impact serrated carcinogenesis by generating a pro-inflammatory microenvironment. Lipopolysaccharide (LPS) is a virulence factor present on *F. nucleatum*, which is recognized by Toll-like receptors to activate the *TLR4/MYD88* pathway, leading to nuclear factor-κB (NF-κB) activation^64^ and release of inflammatory cytokines such as TNF-α, IL-6, IL-8, IL-18.^64,66,74,76,83,84^ IL8 was upregulated in MSI-H CRC.^64^ Inflammation reduces the enzymatic activity of *mismatch repair (MMR)* proteins and causes *MLH1* silencing, leading to MSI.^85^ The potential *F. nucleatum* associated mechanisms involved in the pathogenesis of serrated neoplasm is presented in Figure 3.

## Conclusion and future perspectives

This review summarized the potential association between the gut microbiota and the serrated pathways and proposed putative mechanisms of how gut microorganisms might participate in colorectal carcinogenesis. Although serrated lesions-derived CRC is not the most common type of CRC, its invasiveness and relatively favorable response to target therapy and immunotherapy render it a distinct patient group to be further studied. Most interval cancers in CRCs are proximal tumors with molecular features of *MLH1* methylation, MSI-H, CIMP-H and *BRAF* mutation, and these patients are often diagnosed at advanced stages, with poor prognosis and low survival rates. Early detection of these serrated lesions as premalignant precursors is essential for clinicians. Besides histological and molecular features, the gut microbiota emerges as a critical environmental factor that should be studied to improve the tumor biology, diagnosis, and treatment response of this cancer subtype. Further studies would be necessary to determine the exact role of the gut microbiota in the serrated neoplasia pathway with specific murine models, such as the *BRAF^V637E^* mutant mice,^86,87^ and to identify specific biomarkers for screening, diagnosis, prognosis, and prediction of serrated cancers.

## Abbreviations

CIMP, CpG island methylator phenotype;

CIMP-H, high-level CpG island methylator phenotype;

CIMP-L, low-level CpG island methylator phenotype;

CMSs, consensus molecular subtypes;

CRC, colorectal cancer;

*EGFR, epidermal growth factor receptor*;

EMT, epithelial-mesenchymal-transition;

FadA, *Fusobacterium nucleatum* adhesin;

Fap2, *Fusobacterium nucleatum* autotransporter protein 2;

FFPE, formalin-fixed paraffin-embedded;

FISH, fluorescence in situ hybridization;

Fn-high, high amount of *Fusobacterium nucleatum* DNA in tissues;

Fn-low, low amount of *Fusobacterium nucleatum* DNA in tissues;

GCHP, goblet cell-rich hyperplastic polyps;

*HBEGF, heparin-binding epidermal growth factor-like growth factor*;

HP, hyperplastic polyp;

LPS, lipopolysaccharide;

MDSCs, myeloid-derived immune cells;

*MGMT, O-6-methylguanine-DNA methyltransferase;*

*MLH1, MutL homolog 1;*

*MMP3, matrix metallopeptidase 3;*

*MMR, mismatch repair*;

MSI, microsatellite instability;

MSI-H, high-level MSI;

MSI-L, low-level MSI;

MSS, microsatellite stability;

MVHP, microvascular hyperplastic polyp;

*NF-κB, nuclear factor-κB;*

*PDGRFA+, platelet-derived growth factor receptor alphaþ positive;*

*ROS, reactive oxygen species*;

SAC, serrated adenocarcinoma;

SSL, sessile serrated lesion;

SSL-HGD, sessile serrated lesion with high-grade dysplasia;

TIL, tumor-infiltrating lymphocytes;

TSA, traditional-serrated adenoma;

TSA-HGD, traditional-serrated adenoma with high-grade dysplasia;

TSG, tumor suppressor genes.
